# Unbiased Mitoproteome Analyses Confirm Non-canonical RNA, Expanded Codon Translations

**DOI:** 10.1016/j.csbj.2016.09.004

**Published:** 2016-10-05

**Authors:** Hervé Seligmann

**Affiliations:** Unité de Recherche sur les Maladies Infectieuses et Tropicales Émergentes, Faculté de Médecine, URMITE CNRS-IRD 198 UMER 6236, Université de la Méditerranée, Marseille, France

**Keywords:** Frameshift, Bijective transformation, Digestive enzymes, Unbiased analyses, RNA–DNA difference

## Abstract

Proteomic MS/MS mass spectrometry detections are usually biased towards peptides cleaved by experimentally added digestion enzyme(s). Hence peptides resulting from spontaneous degradation and natural proteolysis usually remain undetected. Previous analyses of tryptic human proteome data (cleavage after K, R) detected non-canonical tryptic peptides translated according to tetra- and pentacodons (codons expanded by silent mono- and dinucleotides), and from transcripts systematically (a) deleting mono-, dinucleotides after trinucleotides (delRNAs), (b) exchanging nucleotides according to 23 bijective transformations. Nine symmetric and fourteen asymmetric nucleotide exchanges (X ↔ Y, e.g. A ↔ C; and X → Y → Z → X, e.g. A → C → G → A) produce swinger RNAs. Here unbiased reanalyses of these proteomic data detect preferentially non-canonical tryptic peptides despite assuming random cleavage. Unbiased analyses couldn't reconstruct experimental tryptic digestion if most detected non-canonical peptides were false positives. Detected non-tryptic non-canonical peptides map preferentially on corresponding, previously described non-canonical transcripts, as for tryptic non-canonical peptides. Hence unbiased analyses independently confirm previous trypsin-biased analyses that showed translations of del- and swinger RNA and expanded codons. Accounting for natural proteolysis completes trypsin-biased mitopeptidome analyses, independently confirms non-canonical transcriptions and translations.

## Highlights

• Unbiased mitoproteomic analyses of trypsinized data confirm trypsin-biased analyses.
• Biased and unbiased analyses confirm non-canonical RNA, expanded codon translations.
• Detected peptides match preferentially vertebrate genetic code translation.
• Previous point excludes cytosolic peptide origins.

## Graphical Abstract

**Figure:**
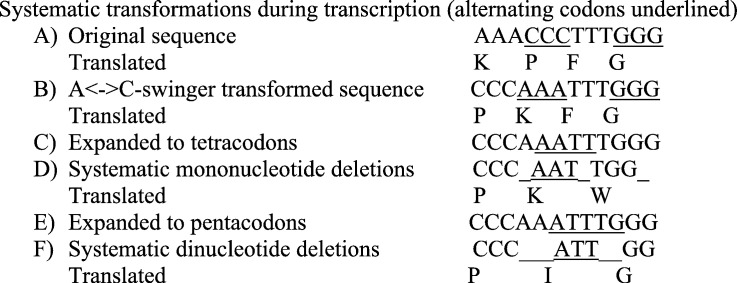

## 1 Introduction

Protein sequences are more complex than texts written in natural human languages [1]. This implies that genes include superimposed information, overprinted on the classical protein coding gene; for example, the three frames of the shortest self-replicating circular RNA virusoid code for proteins [2]. Cryptic coding revealed by frameshifts also implies that a punctuation code regulates ribosomal frame translation. This role seems fulfilled by the natural circular code X, a set of 20 regular codons overrepresented in the coding frame of genes versus other frames. X possesses peculiar mathematical properties that enable retrieval of translational frame [3], [4], [5], [6], [7], [8], [9], [10]. This punctuation code can also be considered as cryptic superimposed information.

The natural circular code seems to prevent unwanted ribosomal frameshifts. In addition, the structure of the genetic code implies a further mechanism against frameshifted translation. This mechanism, rather than preventing ribosomal slippage before it occurs, as assumed for X, minimizes translation after ribosomal frameshifts. This is because the genetic code's codon-amino acid assignments are such that they maximize off frame stop codons [11], avoiding metabolic waste after ribosomal slippages [12], [13], [14], [15], [16].

Superimposed coding is also indicated by other peculiar genetic code properties. The genetic code includes symmetries, such as Rumer's symmetry [17], [18], [19], [20], where transformations of nucleotides into other nucleotides along specific rules reveal symmetries between codon-amino acid families.

Rumer's symmetry implies that after applying that specific transformation, all codons coding for a given amino acid are transformed into codons coding for another amino acid [21]. These theoretical observations seem related to the following empirical observations. Recent transcriptomic and proteomic findings show that genetic information is revealed by each systematic frameshifting and nucleotide transformations. Here I develop these issues and present analyses that strengthen the proteomic evidence for translation of proteins coded by overprinting associated with systematic frameshifts and systematic nucleotide transformations.

**Fig. 1:**
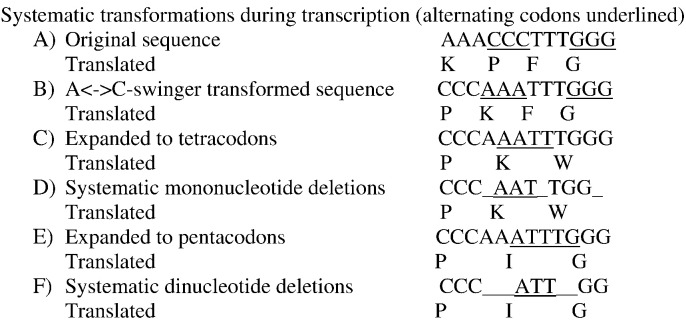
Sequence (A) and its systematic transformations and corresponding translations (B–F). B) A ↔ C systematic nucleotide exchange of sequence in A; C) assuming systematic codon expansion by silent mononucleotides; D) assuming systematic mononucleotide deletion after each trinucleotide (translation identical to that in C); E) assuming systematic codon expansion by silent dinucleotides; F) assuming systematic dinucleotide deletion after each trinucleotide (translation identical to that in E). RNAs and peptides corresponding to these alternative transcriptions and translations have been previously described for human mitochondria [22], [23]. For swinger transformations, A ↔ C is only one among 23 possibilities, nine symmetric of type X ↔ Y, and 14 asymmetric, of type X → Y → Z → X. Systematic deletions of mono- and dinucleotides after each trinucleotide are annotated as delRNA_3–1_ and delRNA_3–2_. Systematic deletions can start at the 5′ extremity of a sequence, which is indicated by delRNA_3–1.0_ and delRNA_3–2.0_, deletion frames can be shifted by 0–2 and 0–3 nucleotides for delRNA_3–1_ and delRNA_3–2_, respectively, which can be indicated by corresponding indices.

Previously detected peptides match predictions of regular translations of non-canonical mitochondrial RNAs, and non-canonical translations of codons expanded by silent mono- and dinucleotides (detailed in Fig. 1). These results seem overall robust as detected non-canonical peptides mapped on the human mitogenome with corresponding non-canonical RNAs [22], [23]. Hence existences of non-canonical RNAs and peptides are validated by independent detections of non-canonical RNAs and peptides, and by convergences (associations) between detected non-canonical RNAs and peptides.

However, MS/MS matching between observed and predicted mass spectra is biased. Hence the unconventional natures of non-canonical transcriptions and translations presumably producing these peptides require careful evaluation of proteomic analyses. Below I review the different types of non-canonical transcriptions and translations, and relevant previous results. Previous conclusions about non-canonical peptides are then re-evaluated according to analyses that account for overfitting that could have affected the previously used proteomic search algorithm [22], [23]. These new analyses strengthen previous conclusions on non-canonical mitochondrial transcriptions and translations.

### 1.1 Non-Canonical Transcriptions: RNA–DNA Differences

Transcription is not always perfectly accurate, but in some cases, RNA–DNA differences (RDDs) are not random and are systematically detected at some specific positions, either in the form of nucleotide substitutions [24], also observed on mitochondrion-encoded RNAs [25], [26], [27]) or deletions [28]. These punctual differences between transcript and DNA occur shortly after transcripts exit the RNA polymerase, suggesting posttranscriptional RNA editing [29]. These single nucleotide modifications produce non-canonical transcripts.

In other types of non-canonical RNAs, modifications occur systematically for all nucleotides for the complete (or almost complete) RNA. Two types of systematic transformations occur: (a) systematic deletions of mono- and dinucleotides after each trinucleotide, producing delRNAs in human mitochondria [22]; and 23 types of systematic exchanges between nucleotides (nine symmetric exchanges, type X ↔ Y, e.g. A ↔ C; and fourteen asymmetric exchanges, type X → Y → Z → X, e.g. A → C → G → A, [30], [31], [32], [33]), producing swinger RNAs.

Swinger- and delRNAs are probably not due to RNA edition, unlike the punctual RDDs. This is because transformations frequently occur systematically on sequences longer than 100 nucleotides. They seem produced by the same RNA polymerase as regular RNA, presumably after the RNA polymerase stabilizes in a hypothetical mode similar to that causing punctual nucleotide misinsertions [32], [33], [34]. This is also indicated by contiguity between regular and swinger sequences in the few detected chimeric RNAs, DNAs and peptides that consist of regular and swinger sequences [35], [36].

### 1.2 Non-canonical Transcriptions: Systematic Deletions

Three independent lines of evidence suggest del-transcription, transcription systematically deleting/jumping mono- and dinucleotides after each trinucleotide. (a) Contiguous short RNA reads in the human transcriptome match the mitogenome transformed by systematic (mono- and dinucleotide) deletions after each trinucleotide [22]. (b) Peptides corresponding to these delRNAs were detected in proteomic data [22]. (c) The human mitogenome, after del-transformations, has more inverted palindromes potentially forming stem-loop hairpins than comparable randomly shuffled sequences [23]. Excess palindromes after del-transformations suggest biological roles for del-transformed sequences. Palindromes in del-transformed sequences apparently down-regulate del-transcriptions [37]. Convergences between these different evidences suggest actual biological roles for del-transformed DNA/RNA.

### 1.3 Non-canonical Transcriptions: Systematic Nucleotide Exchanges

Homology between some RNAs and the ‘parent’ DNA can only be detected when assuming systematic exchanges between nucleotides along the complete RNA length. This process is called ‘swinger’ transcription. Several independent evidences show that swinger polymerizations occasionally occur. (a) Swinger DNA has been detected [39], [40], in addition to swinger RNA [30], [31], [32], [33]. (b) Peptides corresponding to detected swinger RNAs also occur [23]. (c) The human mitogenome includes swinger repeats, meaning repeats of other parts of the mitogenome, at the condition one assumes a given swinger transformation. These swinger repeats are more numerous than for comparable randomized sequences [38], [41]. (d) The swinger-transformed human mitogenome has more inverted repeats potentially forming stem-loop hairpins than randomly shuffled sequences [42]. (e) Chimeric DNA/RNA [35] and peptides [36] exist. These nucleotide and peptide sequences consist of at least two contiguous parts, where one part is ‘regular’ (= untransformed), the other is swinger-transformed according to one of the 23 potential bijective transformations of nucleotide sequences [19], [20].

These various material evidences converge with one another: detected swinger peptides map on detected swinger RNAs [23]; mitochondrial swinger RNA abundances increase with abundances of swinger repeats in the mitogenome; and palindromes formed by swinger-transformed mitosequences associate with swinger RNA detection [42]. This association between transcripts and hairpins for swinger RNA is expected because regular mitochondrial post-transcriptional RNA processing depends on secondary structure formation for regular RNAs, a process called tRNA punctuation of mitochondrial posttranscriptional RNA processing [43]. Hence swinger RNA processing resembles regular RNA processing.

In addition, mitochondrial swinger RNA has been detected within datasets produced by classical Sanger sequencing [30], [31], [32], [33], and by massive next generation Illumina sequencing [23]. Swinger RNA properties converge between RNAs sequenced by these different methods [23].

#### 1.3.1 Systematic Nucleotide Exchanges and the Natural Circular Code

A specific property of the genetic code is that it includes a ‘punctuation’ code which enables retrieval of the protein coding frame, called the natural circular code X [44], [45], putatively by interactions between mRNAs and the ribosomal decoding center [8], [9], [10]. X consists of 20 codons that are over-represented in the coding frame of genes, as compared to non-coding frames, and as a group, have several strong mathematical properties that enable detecting the coding frame.

The 23 bijective transformations (or swinger transformations), when applied to X, produce also circular codes [34], [44]. The reading frame retrieval capacity (RFR) of circular codes can be quantified [5]. The RFRs of these transformations of X correlate with properties of corresponding detected swinger RNAs [34]. This means that strictly theoretical considerations predict swinger transcription properties. Swinger RNA abundances are proportional to the invariance of circular code properties of sequences after corresponding bijective transformations.

Associations between empirical observations of swinger transformations and theoretical properties derived from X are strong evidence that swinger transformations increased the coding potential of short protogenomes. This is because X, shared by almost all organisms [45], is very ancient. Hence swinger transformations were embedded within the polymerization machinery since its earliest inception.

#### 1.3.2 Swinger Transformations and tRNA-Replication Origins

A peculiar observation on palindromes formed by some human mitogenome sequences after specific swinger-transformations also suggests, among others, that swinger transformed sequences are integrated in the genome, and participate in creation of new functional sequences.

Mitochondrial light strand replication typically originates at the OL, the light strand replication origin, a stem-loop hairpin located within the largest tRNA gene group in vertebrate mitogenomes [46]. The OL loop contains the recognition and initial binding site of the mitochondrial DNA polymerase [47], [48]. In several taxa, such as most birds, the OL is totally missing [49], suggesting that its function is performed by adjacent tDNAs, which form OL-like structures [50], [51], [52], [53], [54], [55], [56], [57], [58].

No clear homology between mitochondrial tRNAs and the OL has been observed, despite functional indications suggesting some interchangeabilities between tRNA and OL functions. These include aminoacylation of RNA corresponding to the OL [59] and similarities between tRNA (and tRNA-related enzymes) and elements of the replicational machineries of ancient viruses [60], [61]. Only recent analyses searching for inverted palindromes in the swinger-transformed human mitogenome detected ten nucleotide long complementarity between the human mitochondrial OL loop and the D-arm of mitochondrial tRNA Ala [42].

Eight swinger transformations which form the group 2 bijective transformations [19], create this OL-tRNA palindrome. Hence swinger transformations reveal the previously presumed OL-tRNA homology. This suggests unsuspected evolutionary implications for swinger transformations in the context of *de novo* creation of functional structural RNAs [62]. It also confirms the above considerations that swinger polymerizations occurred since the onset of the molecular machinery of life.

### 1.4 Peptides Matching Translation of Codons Expanded by Silent Mono- and Dinucleotides

Several observations indicate that sequences code for many more proteins than usually assumed. For example, activity of stop-suppressor (or antitermination) tRNAs [63], [64], [65], [66] presumably templated by the antisense sequence of regular mitochondrial tRNAs [55], [65], [66] might enable translation of supposed non-coding frames that include stop codons [67], [68], [69], [70], [71]. This is also suggested by coevolution between predicted mitochondrial suppressor tRNAs and predicted mitochondrial off-frame coding regions in several taxonomic groups (primates [67], [68]; *Drosophila*[68], [69]; turtles [70]; and chaetognatha [71]). These analyses assume a change in genetic code where stop codons are reassigned to code for unknown amino acid(s) [72]. This stop-codon reassignment is also suggested by comparisons between mitochondrial and other genetic codes [72], [73], [74], [75], [76], [77], [78], [79], [80], [81].

Translation by another type of tRNAs, tRNAs with expanded anticodons, unleashes further coding potential. This is indicated by coevolution between predicted mitochondrial tRNAs with expanded anticodons and predicted coding sequences translated from stretches of tetracodons, codons expanded by a silent fourth nucleotide [56], [57], [82], [83].

Presumably, regular tRNA translation of delRNAs produces the same peptides as regular RNAs translated by unusual tRNAs with expanded anticodons [84], [85], [86], [87], [88], [89], [90]. Expanded codons are compatible with symmetry and error-correcting properties of the tessera, a subset of 64 among the 264 tetracodons. Tessera are the presumed ancestors of the vertebrate mitochondrial genetic code [91]. The tessera hypothesis is compatible with the fact that regular codon–anticodon interactions are too weak for peptide elongation without ribosome, which presumably evolved after primordial translation mechanisms [92], [93], [94].

Some mitochondrial peptides match translations according to tetra- and pentacodons, including for translations of swinger-transformed versions of the human mitogenome [22], [23]. This type of peptide translation is particularly peculiar. For now it is deduced from (a) coevolution between predicted tRNAs with expanded anticodons with predicted tetracoding sequences, (b) empirical matches between predicted and observed MS/MS spectrometry data, and (c) associations between detected peptides and corresponding detected non-canonical swinger RNAs. Fig. 1 shows examples of translation according to tetra- and pentacodons. Hence re-analyses are designed to avoid some biases present in previous analyses. These reanalyses confirm the validity of previously described non-canonical peptides, particularly those coded by expanded codons [22], [23].

### 1.5 Supervised versus Unsupervised Analyses

Proteomic analyses characterize protein expression patterns from mass spectrometry data of cell proteome extracts. These typically match numerous MS/MS spectra predicted from the annotated genes in genomes with observed spectra (e.g. for the bacterium *Tropheryma whipplei*, agent of Whipple's disease [95]). This approach is biased: it optimizes the fit between observed and expected MS/MS datasets. Such supervised/biased analyses always imply some false positive detections due to overfitting between observation and prediction, particularly for large datasets [96], including microarray analyses [97], [98], [99]. Deliberate biases in analyses also improve estimations [100], but overfitting remains a problem, especially for detection of unknown phenomena.

A known bias that affects classical proteomic search algorithms is that predicted protein sequences are matched to observed data, assuming specific cleavage according to cleavage by the digestion enzyme used during protein/proteome extraction and preparation. Hence if trypsin was used during sample preparation, the amino acid at the carboxyl extremity of peptides is *a priori* supposed tryptic, specific cleavage after K or R.

Hence searches are usually biased towards peptides matching the cleavage rules of digestion enzyme(s) used during sample preparation, because this limits greatly cleavage options, saves computational machine time. Detections of non-canonical peptides would be validated if unsupervised analyses that do not predefine specific cleavage rule(s) detect mainly tryptic peptides. Unbiased analyses can only recover experimental tryptic conditions if most non-canonical peptides detections are accurate.

Proteomic search algorithms usually enable analyses assuming random cleavage when fitting observed and expected mass spectra, by options indicating ‘no enzyme’ or ‘no specific cleavage’. This option is rarely used, because it increases search times enormously.

Here analyses assume random cleavage of actually tryptic proteomes. These should preferentially detect peptides ending by K or R, as compared to other amino acids. This biased result for unbiased analyses would validate conclusions from previous trypsin-biased analyses. The latter detected numerous peptides matching non-canonical RNAs and translations [22], [23]. Here I aim at confirming these previous results using unbiased analyses assuming random cleavage.

### 1.6 Unsupervised Analyses and Natural Proteolysis

Numerous natural proteases are active in cells, including in mitochondria, forming the mitodegradome [101]. Natural proteolysis interferes with proteomic analyses based on artificial additions of digestion enzymes [102], [103], [104]. Analyses accounting for natural proteolysis can complete proteome descriptions [105], [106], [107], [108], [109], [110], [111]. Hence unsupervised analyses assuming random cleavage might detect some actual non-tryptic peptides produced by natural proteolysis or spontaneous protein degradation (especially during sample preparation), potentially completing descriptions of non-canonical mitochondrial peptidomes.

Analyses examining associations between non-tryptic non-canonical peptides and previously detected corresponding non-canonical RNAs [22], [23] could test whether non-tryptic peptides are false positives. Positive results would validate the existence of non-canonical transcriptions and translations, independently of the expected bias for tryptic peptides among peptide populations detected by unbiased analyses.

### 1.7 Hypotheses and Predictions

Here unbiased proteomic analyses search tryptic human proteome data [112] for peptides matching translations of del- and swinger-transformed versions of the human mitogenome, as done by previous biased analyses that assumed tryptic digestion [22], [23]. Unbiased analyses assume random protein cleavage. They are applied to the same proteomic data as previous tryptic-biased analyses that detected non-canonical peptides that match del- and swinger-transformed versions of the human mitogenome (the latter according to three codon sizes, tri-, tetra- and pentacodons). Properties of detected non-canonical peptides are compared to those detected by classical, trypsin-biased analyses.

The working hypothesis predicts that unbiased analyses detect peptide populations biased towards trypsin-digestion. This result would mean that non-canonical peptides are not false positives. Unbiased analyses could not reconstruct tryptic experimental conditions unless a majority of detected peptides were true detection. The second aspect of the working hypothesis is that detected non-tryptic non-canonical peptides result from natural proteolysis, and hence are not false positives. In that case, these should map preferentially on detected non-canonical RNAs, as previously observed for tryptic non-canonical peptides.

The primary aim is to test whether conclusions from previous results obtained by trypsin-biased analyses can be qualitatively reproduced by unbiased (unsupervised) analyses, considering potential natural proteolysis/spontaneous protein degradation, rather than experimentally added trypsin. Confirming natural proteolysis and expanding the coverage of the non-canonical mitoproteome are secondary aims. Analyses are restricted to predictions of peptides encoded by the mitogenome and its various systematic del- and swinger transformations.

## 2 Materials and Methods

Materials and methods are essentially identical to the corresponding sections for peptides translated from del-transformed versions of the human mitogenome [22], and those translated from the swinger-transformed versions of the human mitogenome [23]. The only difference is in the fact that the proteomic search software Proteome Discoverer 1.3 (Thermo Fisher Scientific, Illkirch) is set to analyze proteomic data digested by ‘no enzyme’. The same data as previously are analyzed [112].

As for previous analyses [22], [23], associations between detected non-canonical peptides and corresponding detected non-canonical RNAs are based on human transcriptomic data [113], as previously presented (del-RNAs [22], therein Table 1, Table 2; swinger-RNAs [23], therein Table 1 and supplement). For swinger-transformed versions of the human mitogenome, predicted peptides are translated according to each tri-, tetra- and pentacodons, as previously described [22], [23].

All frames of transformed sequences were translated according to the vertebrate genetic code, three, four and five frames for each positive and negative strands, for codon sizes three, four and five, respectively. For codon sizes above three, codons are translated according to the genetic code, expanding the codon by silent mono- and dinucleotides, respectively. The next codon in these cases does not include the silent nucleotide(s) (see Fig. 1). The hypothetical peptides translated from non-canonical mitogenome transformations and along non-canonical codon sizes were trypsinized *in silico*, to create the fasta file containing predicted peptides.

Stop codons are translated by the letter ‘X’, which the software Proteome Discoverer recognizes as Leu or Ile (not distinguishable by mass spectrometry because of equal masses). Each predicted peptide including at least one stop is represented 19 times in the input database of hypothetical predicted peptides, replacing all stops by one among the 18 remaining amino acid species, excluding Leu and Ile.

Consensus searches were handled with the Sequest (Thermo Fisher Scientific, Illkirch) algorithm with molecular mass tolerances: Parent = 1 Da and Fragment = 0.5 Da (monoisotopic masses). I activated fixed carbamidomethyl (C) and variable Oxydation (M) modifications, as well as the lysine → pyrrolysine modification.

### 2.1 Why Include Lysine to Pyrrolysine Modifications?

An anonymous reviewer notes that pyrrolysine is not a lysine modification, but is usually encoded by UAG stop codons [114], [115], [116]. It is presumably not encoded in eukaryotes. There are several reasons for allowing lysine → pyrrolysine modifications, despite that this probably increases search times. The first reason is methodological: results of the present analyses have to be comparable to previous searches, which allowed this modification. The second reason is that non-canonical peptides might result from mechanisms that are relicts from the mitochondrion's bacterial ancestors, which probably did translate UAG by pyrrolysine.

Hence allowing this modification might enable detecting peptides that otherwise would not be detected, when stops are assumed translated by lysine. The software does not differentiate between ‘regular’ lysine and lysine translated by stops. Analyses presented here do not explore issues implied by modifications, but presented data include that information for future analyses.

### 2.2 Unbiased Analyses Can't Include Nucleus-Encoded Proteins

This anonymous reviewer also indicates that analyses should ideally include the predicted canonical human nuclear-encoded proteome, including the mitochondrial nuclear-encoded proteins imported from the cytosol. These canonical proteins would provide valuable controls for analyses designed to detect non-canonical peptides.

First, they would prevent spurious matches between observed mass spectra and predicted non-canonical peptides resembling canonical peptides. Secondly, one expects much fewer detections of non-canonical than canonical peptides, an additional prediction that can be tested. Third, such analyses would enable to test the hypothesis that higher proportions of non-canonical than canonical peptides are non-tryptic (versus tryptic ones). This hypothesis assumes directed natural proteolysis of non-canonical, hence probably dysfunctional, peptides.

The first point is handled by a different, less time-consuming analysis described in the Results section, which shows that such spurious results are unlikely. Unfortunately, the nucleus-enocoded canonical proteome can't be included in unbiased analyses. This is not only because results from unbiased analyses have to be compared to previous biased analyses that did not include the canonical nucleus-encoded proteome (reanalyses including them are planned).

Unbiased analyses including the much larger canonical nucleus-encoded proteome are technically impossible with available computing capacities. Including these canonical proteins would manifold increase numbers of predicted peptides to be matched with observed mass spectra. This would render analyses impractical, to unknown extents, as searches excluding canonical nucleus-encoded proteins last 10 days. Hence inclusion of these controls will have to wait for commercial availability of computers and software with parallel processing capacities greater than those used now (I use a machine that has 32 parallel processors, regular PCs have 2 processors). It is adequate to remind here that analyses reported here are already control analyses for previous results. Hence inclusion of further controls, though valuable, has always an arbitrary component, besides the above noted technical problems.

### 2.3 Peptide Detection Criteria

False discovery rates FDR [117], [118], [119] were estimated against a reverse decoy database using the Percolator algorithm. No protein grouping was allowed since the database only contained non-redundant entries. Peptides are considered detected with FDR q < 0.05 and Xcorr > 1.99. FDR is calculated by comparing Xcorr obtained from expected and observed MS/MS mass spectra with those obtained for a decoy database of false negative predicted peptides.

Xcorr is a cross-correlation statistic that compares observed and predicted MS/MS data. It sums the products between observed (y) and expected (x) values for series of data. In this case, these are the observed and expected mass spectrometry data [120]:

$$\text{Xcorr}=\sum_{i=0}^{n-1}x\left(i\right)*y\left(i+\tau \right),$$

where τ is a displacement (lag) between observed and expected data for position i, with n positions in the data.

Peptide posterior error probabilities (PEP) are also indicated. PEP estimates confidence in detections of specific individual peptides. This approach differs from q, designed to estimate confidence for groups of detected peptides. The latter optimizes between false positive and false negative rates. PEP should be used with caution because it inflates false negatives [117]. Analyses focus on peptide populations and hence do not integrate PEP, but PEP is indicated because it could be useful in the context of future analyses focusing on specific peptides.

## 3 Results

### 3.1 Unsupervised Analyses Assuming Random Cleavage

Proteomic analyses of the 96 human proteome extracts [112], when no specific cleavage enzyme is specified, lasted about 10 days for each unsupervised analysis. Four such unbiased 10-day long searches were performed, for peptides matching translations of the nine del-transformations of the human mitogenome (four del-transformations deleting a mononucleotide after each trinucleotide, and five del-transformations, deleting a dinucleotide after each trinucleotide), and of the 23 swinger-transformed versions of the human mitogenome. The latter are translated according to three codon sizes: regular tricodons, tetra- and pentacodons, which are regular codons expanded by silent mono- and dinucleotides.

Comparable analyses of these data and predicted peptides, but trypsin-biased, last 8–9 h for each analysis. The longer times required for analyses reflect much greater potential cleavage combinations when comparing observed and expected peptide mass spectra for unbiased analyses.

#### 3.1.1 Unsupervised Analyses: Bias for Tryptic Peptides

All four unsupervised searches matching observed MS/MS mass spectra with predicted ones detected preferentially tryptic peptides (carboxyl-extremity K or R, Table 1). Hence without *a priori* biasing searches, populations of detected non-canonical peptides are for these analyses biased towards tryptic peptides.

Biases for residue identity at the carboxyl extremity of detected peptides are calculated as the frequency of observing a given amino acid at that position in detected peptides, divided by that amino acid's frequency in hypothetical peptides translated from the corresponding complete transformed human mitogenome. The highest bias favors lysine (K) for translation of delRNAs, swinger RNAs, and swinger RNAs according to pentacodons, and second highest (after bias for Q) for swinger RNAs according to tetracodons. Lysine is one among 19 possibilities, so the probability to obtain the strongest bias for K is 1/19 = 0.053, for tetracodons the result has P = 0.11.

According to Fisher's method for combining independent P values [121], the overall result for bias in favor of K across all four analyses is P = 0.0046. Hence results show a strong bias favoring K at the end of detected peptides, despite assuming random cleavage.

Bias for arginine (R) at the carboxyl extremity of detected peptides is the second highest for peptides matching del-transformed versions of the human mitogenome (excluding K, this has P = 1/18 = 0.056), the fourth highest for regular translations of the 23 swinger-transformed versions of the human mitogenome (P = 0.17), and the third highest for their translation according to tetra- and pentacodons (P = 0.11, each). According to Fisher's method for combining independent P values [121], the overall result for bias in favor of K across all four analyses is P = 0.02.

Bias for combined K and R ending peptides is highly significant according to chi-square tests for each of the four independent analyses: delRNAs, P = 0.00023; swinger RNAs according to tricodons, P = 0.0016; tetracodons, P = 0.00000006; and pentacodons, P = 0.000038.

This means that unsupervised searches for non-canonical peptides detect preferentially non-canonical peptides that match the known tryptic sample preparation. This result could not be obtained if the majority of detections were false positives. Hence these biased results obtained from unbiased analyses confirm that the various populations of non-canonical peptides (peptides translated from delRNAs, and from swinger RNAs, these translated according to regular tri-, tetra and pentacodons) exist. This result is not trivial, and independently confirms conclusions from previous trypsin-biased analyses [22], [23].

### 3.2 Search Bias and Absolute Versus Relative Majority of Tryptic Peptides

An anonymous reviewer notes that tryptic peptides are only a relative majority among detected peptides, rather than an absolute majority. This might to some extent contradict the above conclusion of bias towards tryptic peptides. This issue can be understood by comparing the tryptic bias obtained from unbiased analyses, to that obtained for chymotrypsin-biased analyses.

These chymotrypsin-biased analyses were for the same proteomic data and the same predicted non-canonical peptides ([122], therein supplementary data). They differ from previous analyses assuming tryptic digestion, and the current unbiased ones, because analyses assumed chymotryptic digestion. Analyses assuming chymotryptic digestion detected 479 non-canonical peptides, among which 131 (27.35%) had carboxyl terminal residues matching chymotryptic digestion (W, Y and F). All other peptides (72.65%) were tryptic. Hence the absolute majority of peptides detected by these analyses biased towards three possible non-tryptic carboxyl terminal residues are tryptic peptides, as expected.

These chymotrypsin-biased analyses were completed by three separate analyses biased to detect only peptides with a specific, chymotryptic ending, hence separately W, Y, or F. In these analyses set to detect peptides with only one possible chymotryptic residue at its carboxyl terminus, tryptic peptides represent on average across all analyses 87.3% of all detected peptides.

In order to compare these results with those from unbiased analyses presented here, I calculated the bias between tryptic peptides and peptides matching each other possible (non-tryptic) carboxyl terminus for data in Table 1. Tryptic peptides are on average 82.76% of the peptides in Table 1 when considering only one alternative residue at the carboxyl terminus. This average value is very comparable to the above mentioned 87.3% tryptic peptide majority obtained for chymotrypsin-biased analyses searching for only one of the three chymotryptic carboxyl termini.

The meaning of this is mathematically trivial. Tryptic bias decreases the more other options are allowed. It is highest when analyses are biased towards tryptic peptides: all detected peptides were tryptic. This bias decreases when analyses are biased towards a single different possibility (separating W, Y and F). Tryptic bias further decreases when all three chymotryptic carboxyl termini are considered (W, Y and F). Tryptic bias is lowest, yet still statistically significant, when analyses are unbiased, as when considering all results in Table 1. When averaging abundances of non-tryptic carboxyl termini in Table 1, the tryptic bias is comparable to that obtained for analyses biased towards only one non-tryptic carboxyl terminus, as obtained for analyses biased separately towards W, Y or F.

The issue of relative versus absolute tryptic majority is only a matter of doing adequate comparisons. I agree that adding canonical nucleus-encoded proteins in the analyses would probably yield valuable further insights in this context, regarding potential biases for tryptic canonical peptides, versus more non-tryptic natural digestions for non-canonical peptides. Currently such analyses are technically impossible in the context of unbiased analyses.

### 3.3 Associations between Non-canonical RNA and Peptide Abundances

Peptides with non-tryptic carboxyl extremity could represent false positive detections. They might alternatively result from natural proteolysis/spontaneous degradation of proteins. The bias for tryptic peptides (previous section) corresponds to experimental trypsin-preparation of extracts. Remaining non-tryptic peptides are not necessarily false positives.

This can be tested by exploring associations between non-canonical peptides and corresponding non-canonical RNAs, as previously described for trypsin-biased analyses [22], [23]. Two independent methods are used in this respect: (a) Pearson correlation analyses between abundances of detected non-canonical peptides and corresponding RNAs, expecting positive correlations between abundances; (b) precise mapping of individual peptides and RNAs, which expects that more detected peptides map on detected RNAs than expected by chance. These positive associations between non-canonical peptides and RNAs would show the regular causal link between RNA and peptides, for the various non-canonical transcriptions and translations.

Numbers of non-canonical peptides are counted from lists of peptides in the supplementary data, mitogenome coverages by non-canonical RNA for delRNAs and for swinger RNAs are from previous publications (delRNAs, [22], therein Table 1, Table 2, for systematic mono- and dinucleotide deletions, respectively; swinger RNAs [23], therein Table 1). These non-canonical RNAs had been detected from human transcriptome data [113], using blastn with Megablast default alignment parameters [123].

For delRNAs, correlation analyses of associations between abundances depend on nine observations, based on coverages of the del-transformed human mitogenome. There are four and five observations, according to the four frames of systematic mononucleotide deletions, and the five frames of systematic dinucleotide deletions, respectively (data in [22], therein Table 1, Table 2). Correlation analyses for swinger transformations are based on 23 observations, for each swinger transformation of the human mitogenome (RNA coverage data from [23], therein Table 1).

For tryptic peptides detected by unbiased analyses, abundances of detected swinger peptides coded by regular codons are proportional to corresponding swinger RNA coverage of the human mitogenome (r = 0.45, P = 0.016, one tailed test). Correlations are positive but not statistically significant at P < 0.05 for swinger analyses of other codon sizes, and del-transformations. Combining the four P values using Fisher's method for combining P values [121] yields an overall significant positive association (P = 0.026). These results from unbiased analyses confirm previous trypsin-biased analyses [22], [23].

Correlation analyses for non-tryptic peptides detected by unbiased analyses are also positive, though never statistically significant at P < 0.05, also not after combining P values (P = 0.104). These weaker associations between peptide abundances and RNA coverage for non-tryptic peptides suggest that a greater proportion of these peptides could be false positive detections, though the overall positive trends are rather compatible with them resulting from natural proteolysis. Local mappings on RNAs below test this point.

### 3.4 Local Mapping of Non-canonical RNA and Peptides

Some peptides detected by unsupervised proteomic analyses map on previously detected, corresponding non-canonical RNAs (Table 1). Previous similar analyses for non-canonical peptides detected by trypsin-biased searches showed that detected peptides map more frequently than expected by chance on corresponding detected non-canonical RNAs, for del- and swinger-transformations [22], [23].

Analyses across unsupervised analyses for del- and swinger-transformations find that 4 among 102 detected non-canonical tryptic peptides (3.9%) map on previously detected non-canonical RNAs. Only 2.64 among these 102 detected tryptic peptides should map on detected RNAs if mapping is random. Small sample size does not enable statistical testing, but suggests a non-significant difference corresponding to the expected association between non-canonical RNAs and peptides. Though this result is not statistically significant, it should be considered as confirmative as it is in line with previous, statistically significant results for tryptic peptides detected by analyses biased towards tryptic peptides.

For non-tryptic peptides detected by unsupervised analyses, 29 among 388 (7.5%) map on previously detected RNAs. This is statistically significantly more than the 13.01 expected according to random mapping (chi-square test, P = 0.000009). Rates of mapping on RNAs do not differ between tryptic and non-tryptic peptides according to a chi-square test. Hence overall, there are not more false positive detections of non-tryptic than tryptic peptides.

In total, 33 among the 490 non-canonical peptides detected by unbiased analyses map on non-canonical RNAs (6.7%), which is statistically significantly more than the 15.65 expected by chance (chi-square test, P = 0.000012).

These results for non-tryptic peptides detected by unbiased analyses confirm conclusions about non-canonical transcriptions and translations, independently of previous results for tryptic peptides detected by trypsin-biased proteomic analyses. Notably, results from independent analyses strengthen conclusions that swinger RNAs are also translated according to tetra- and pentacodons.

### 3.5 Unique Versus Multiple Detections of Tryptic Peptides

Unbiased analyses confirm previous trypsin-biased analyses in two ways. First, they detect preferentially tryptic peptides. This corresponds to the tryptic experimental design. Second, detected peptides associate with previously detected RNAs, for tryptic and other peptides, as found in previous publications for tryptic peptides detected by trypsin-biased analyses [22], [23].

These results independently confirm trypsin-biased analyses because most tryptic peptides detected by trypsin-biased analyses differ from those detected by the unbiased analyses presented here. Only some tryptic peptides detected by trypsin-biased analyses are also detected by unbiased analyses (one or two peptides). Hence analyses confirm independently of previous analyses, the previous results for trypsin-biased analyses.

Note that analyses of the same data, testing the same hypotheses, and assuming chymotryptic digestion, yield similar conclusions. These analyses detect majorities of tryptic peptides. Both tryptic and chymotryptic peptides associate with detected RNAs [122]. Hence unbiased analyses yield a third independent confirmation of results obtained by tryptic- and chymotryptic-biased analyses.

### 3.6 Negative Control: Residues after the Carboxyl-Terminal of Detected Peptides

A further analysis shows a peculiar unknown fact about natural mitochondrial proteolysis. Unbiased analyses yield peptide populations with diverse residues at their carboxyl extremity, which might mainly reflect proteolysis by naturally occurring digestion enzymes in human mitochondria. The alternative hypothesis (or rather the null hypothesis) is that peptides were actually randomly cleaved, which would also be compatible with random peptide detections, possibly due to majorities of false detections.

Biases for tryptic peptides overall falsify the random cleavage hypothesis, but remaining peptide populations, after excluding tryptic peptides, might nevertheless fit random cleavage. Here control analyses test for this by recording the first amino acid expected according to non-canonical translations, after the carboxyl extremity of the detected peptides. According to the compilation by ExPaSy PeptideCutter (http://web.expasy.org/peptide_cutter/peptidecutter_enzymes.html, accessed 6VI2016), the majority of listed specific cleavage rules relate to the carboxyl extremity of peptides, rather than the N-terminal of the next peptide. Nevertheless, the possibility that populations of detected peptides include biases for N-terminal cleavage in relation to the ‘downstream’-encoded amino acid is plausible.

Table 3 presents biases, calculated as for Table 1 by using total abundances of amino acids, for amino acids at the N-terminal of the peptide located after the detected peptides, for the various unsupervised analyses (peptides translated from del- and swinger-transformed human mitogenome, and translated according to tetra- and pentacodons for the swinger-transformed versions).

These biases do not resemble biases detected for the carboxyl extremity of the detected peptides, when considering the same amino acid species. Bias distributions are systematically less extreme for N-terminals of the next undetected peptide than for the carboxyl extremity of detected peptides. For the N-terminal of the next peptide, the lowest bias is 0.44, 0.40, 0.33 and 0.42 for detected peptides translated from del-, swinger-transformed versions of the human mitogenome, and for swinger-transformed mitogenome translations according to tetra- and pentacodons. Such biases below “1” indicate cleavage avoidance. For the carboxyl terminal of detected peptides, corresponding minimal biases are 0 for each non-canonical translation, the strongest possible negative bias.

Maximal biases for N-terminals of undetected peptides next to detected peptides are 1.59, 1.98, 1.64 and 2.31. For carboxyl extremities of detected peptides, corresponding maximal biases are 2.43, 2.29, 3.15 and 3.22. Overall, distributions for biases of amino acid identities at the N-terminal of next peptides are much closer to the value ‘1’, indicating no bias, and seem random around this value. This suggests that there is no evidence for N-terminal specific cleavage in these data for the human mitochondrial proteome.

These analyses show non-random patterns in cleavages for detected non-canonical peptide populations, for carboxyl termini. In this respect, results for N-termini function as negative controls and strengthen confidence in results.

### 3.7 Few Nuclear Contaminations: Peptides Follow the Mitochondrial Vertebrate Code

Eukaryotic nuclear genomes include numerous inserts of the mitogenome. Hence detected non-canonical peptides could originate from non-canonical transcriptions and translations of such nuclear mitogenome inserts, or from translations of nuclear sequences that by chance resemble the transformed mitogenome. This possibility is tested by translating the transformed mitogenome using the nuclear genetic code, and by checking whether detected non-canonical peptides are compatible with translation according to the nuclear genetic code.

Considering that coding assignments of 60 among 64 codons (93.75%) are identical for the nuclear and the vertebrate mitochondrial genetic codes, I calculated numbers of peptides, considering lengths of detected peptides, expected to match also nuclear genetic code translation. I used equation N × 0.9375^− k^, where N is the number of detected peptides with k residues. This equation expresses the fact that all codons coding for the peptide must be among those invariant between the two genetic codes, when one or more codons belong to the four codons differing between these two genetic codes, the detected peptide is incompatible with the nuclear genetic code.

There are 177.86 peptides expected compatible with both codes across all analyses. This is far more than the 91 detected non-canonical peptides with translations identical according to both genetic codes. Comparisons between expected and observed peptides compatible with translation according to the nuclear genetic code, separately according for the four different non-canonical transcriptions and translations, follow the same principle: observed numbers of peptides compatible also with the nuclear genetic code are far fewer than expected (Table 4).

This bias means that observed non-canonical peptides match specifically more than expected by chance translation according to the mitochondrial vertebrate genetic code. This result also excludes that detections of non-canonical peptides are incorrect, that these mass spectra actually correspond to similar, nucleus-encoded canonical peptides. This is because the analysis reported in this section accounts for the extreme and plausible situation where sequences identical to the mitogenome were translated. The fact that analyses differentiate between nuclear versus mitochondrial translations of the mitogenome is incompatible with nuclear contaminations.

## 4 General Discussion

Analyses presented here are mainly designed to test conclusions from previous analyses of the human mitochondrial peptidome (data from [112]), where non-canonical peptides matching translations of del- and swinger-transformed versions of the human mitogenome were detected, including translations of expanded codons. Del-transformations assume transcription that systematically deletes mono- and dinucleotides after every third transcribed nucleotide [22]. Swinger-transformed RNAs result presumably from systematic nucleotide exchanges, during transcription along 23 exchange rules, also called bijective transformations [34]. The human proteome includes peptides matching detected swinger RNA, translated according to tri-, tetra- and pentacodons (expanded by silent mono- and dinucleotides) [23].

These previous analyses assumed tryptic proteome preparation [112]. Hence the first set of analyses was biased by information corresponding to sample preparation. Here analyses of the same data were repeated without using that information on tryptic-digestion, but assuming random cleavage. Results indicate a positive bias towards detection of tryptic non-canonical peptides by unsupervised analyses. This result is a strong confirmation that overall, populations of detected non-canonical peptides are not false positives: otherwise, unbiased analyses would not detect positive bias for tryptic peptides. This implies that these non-canonical transcriptions and translations are a biological reality.

Results of unbiased analyses also suggest the possibility that the proteome underwent other specific cleavages, presumably resulting from natural proteolytic activity in the biological sample, such as described for chymotrypsin [116]. Overall, tryptic and non-tryptic non-canonical peptides associate with previously detected corresponding non-canonical RNAs [22], [23], [116]. Convergences between peptide and RNA detections are further evidence that overall, tryptic and other detected peptides are not false positives.

In addition, detected non-canonical peptides preferentially match translation according to the vertebrate mitochondrial genetic code: fewer than expected by chance are compatible with translation according to the nuclear genetic code, considering that 93.75% of codons follow the same translation rules according to both genetic codes. This result is incompatible with detection of peptides originating from the cytosol, even for nuclear DNA sequences identical to the mitogenome.

### 4.1 Statistical Considerations and Peptide Detection

One can argue that non-canonical peptides were detected by chance and are false positives, due to a very large number of comparisons between predicted peptides and observed mass spectra. If it was so, (a) peptide detections would not be biased towards independently detected RNAs, (b) towards translation specific to the vertebrate mitochondrial genetic code, and (c) peptide populations detected by unsupervised analyses would not be biased towards experimental tryptic cleavage. In addition, peptide detections are confirmed by false detection rates q, based on decoy peptides that function as negative controls. FDR takes into account sample sizes (as do usual P values), but also the number of statistical tests done.

The last point in this argument is because analyses account that at stops, every possible amino acid could be inserted. Hence matching observed and expected peptides based on their molecular weight is not sufficient to ascertain the sequence of the peptide: the program can adjust any MS/MS spectrum with a close weight to one of the 19 peptides produced by sequences including at least one stop.

This point does not consider that MS/MS spectrometry accounts not only for total mass, but also for masses of secondary fragments. The simple example of peptide EFG can be helpful here. EFG has the same molecular weight as peptides EGF, FEG, FGE, GEF and GFE and can't be differentiated from these five other peptides by its total mass. However, the estimate of that mass is typically combined with estimates of secondary fragments. Only two peptides, EFG and GEF are compatible with detection of the mass of EF. The same point is valid for observing a mass corresponding to FG, which is also compatible with two peptides, EFG and FGE. The combined observation of masses corresponding to these two fragments characterizes the entire peptide sequence.

In addition, mass spectrometry analyses consider separately b and y ions. Hence the same sequence characterization may occur independently according to both ion types. The score Xcorr integrates these pieces of information, and is the statistic on base of which FDR is calculated to minimize false positives. In fact, numerous tryptic peptides were not detected by original analyses assuming that tryptic digestion was detected by analyses assuming non-tryptic digestion. In addition, previous analyses showed that tryptic peptides detected twice, by analyses assuming tryptic and chymotryptic digestions do not differ in detection accuracy from those detected only by one of these analyses [116]. This suggests that the methodology used for peptide detections is rather prone to false negatives, rather than false positives. False positives are probably a small minority reduced to few individual cases that would not qualitatively alter conclusions.

### 4.2 Potential Confounding Factors: Nuclear Contaminations

The first detected swinger-transformed sequences are RNA and DNA sequences in Genbank's databases (EST data for RNA) longer than 100 nucleotides. These were detected by blast using default megablast alignment search parameters for input sequences consisting of *in silico* swinger transformed mitogenome versions [30], [31], [32], [33]. The detected GenBank sequences aligning with high identity levels with *in silico* produced swinger mitogenome versions (> 90% identity) were sequenced by the classical Sanger technology. Similar searches in Genbank's human transcriptome SRA (sequence read archives) data produced by RNA seq (Illumina) next generation sequencing technology using blastn (also with default search parameters) confirmed the relative abundances of swinger RNAs [23].

Further megablast analyses could not detect alignments between any human nuclear chromosome sequence and del-, swinger-transformed mitogenome versions. However, blastn analyses detected such alignments that could potentially confound several alignments between the transformed mitogenome and RNA seq data. Nevertheless, the majority of RNA seq alignments are due to RNAs originating from the mitochondrion, and are not nuclear, because identities between the transformed mitogenome versions and RNA seq sequences are greater than with corresponding nuclear chromosome sequences, and this for each del- and swinger-transformed mitogenomes [22], [23].

Note that nuclear chromosome sequence alignments with the del- and swinger-transformed mitogenome imply that besides regular mitochondrial mitogenome inserts in nuclear chromosomes (numts, [124], [125], [126], [127], [128], [129], [130], [131], [132], [133], [134], [135], [136], [137], [138], [139], [140], [141]), transformed versions of the mitogenome (or part of) occur in the nuclear genome. Alternatively, regular numts are transcribed according to del and swinger non-canonical systematic transformations. At this point, the main issue is the existence of polymerizations producing systematic transformations, independently of cell compartment where these occur, or whether produced by replication, reverse transcription or transcription. Hence answering with certitude to these questions beyond explained above, though important, is secondary at this point.

In addition, nuclear contaminations are at most minor for peptides presented here, because detected non-canonical peptides are less frequently compatible with both nuclear and vertebrate mitochondrial genetic codes than expected by chance. This bias suggests high specificity for mitochondrial origin of detected peptides.

### 4.3 Potential Confounding Factors: Heteroplasmy

Heteroplasmy [142], [143], [144] is a further known phenomenon that could explain results. However, single nucleotide substitutions can't explain observations of long, non-canonical peptides. Hence only length heteroplasmies, especially those resulting from insertions, could by chance explain non-canonical peptides predicted from systematic mitogenome transformations.

However, the most common length heteroplasmies are relatively few and mainly located in the mitochondrial control region [145], while the peptides detected for the various non-canonical transcriptions and translations are distributed all around the mitogenome. This excludes length heteroplasmies as a major confounding factor for detections of non-canonical peptides.

### 4.4 Potential Confounding Factors: Fused Transcripts

Some transcripts result from fusion of RNA transcribed from DNA regions that are not contiguous [146], [147]. This can result from reverse-transcription artifacts during cDNA production [148]. Fused swinger RNAs also exist [35]. Fusions of regular RNAs are unlikely to produce RNAs that would mimick products of systematically transforming transcriptions. Hence only few single detected non-canonical peptides could by chance correspond to RNA fusions. Artificial transcript fusions during cDNA production could not have produced detected peptides.

### 4.5 Natural Proteolysis of Canonical Versus Non-canonical Peptides

An anonymous reviewer suggested that proteomic analyses should include classical, canonical proteins. This would enable comparing results between canonical and non-canonical peptides, expecting fewer non-canonical peptides than canonical ones. In addition, the reviewer expected that non-canonical peptides would more frequently match non-tryptic, hence natural proteolysis, than canonical peptides. The rationale behind this prediction is that one could expect that non-canonical products are preferentially digested as waste than products of canonical genes.

Practical reasons prevented me from performing these tests. These additional analyses require including among predicted peptides the complete human proteome (corresponding to more than 20,000 genes). This increases numbers of predicted peptides to extents that, for unbiased analyses, are incompatible with current computing capacities. For these reasons, previous and current analyses have been restricted to peptides encoded by the human mitogenome, excluding nucleus-encoded mitochondrial proteins, which are imported from the cytosol into the mitochondrion [149], [150], [151], [152].

A possible solution to this technical problem is to sample the canonical proteome. Analyses searching for peptides matching the swinger-transformed versions of the human mitogenome, translated according to regular tricodons, included such a control. These analyses included peptides predicted according to the regular translation of the untransformed human mitogenome, with the canonical mitochondrion-encoded genes. Fifteen among the detected peptides correspond to translation of the untransformed human mitogenome, among which a single tryptic peptide (6.7% of detected peptides encoded by the untransformed mitogenome). However, 20.9% of the remaining non-canonical peptides are tryptic.

This difference is not compatible with the hypothesis that natural proteolysis digests preferentially non-canonical peptides. However, this qualitative result is not statistically significant, due to small sample size. In addition, the fifteen peptides translated from the regular mitogenome are not restricted to canonical translation of the 13 proteins encoded by the human mitogenome. They include translations of other frames of these genes, and of other sequences (e.g. rRNAs etc). This hypothesis requires analyses specifically designed to test its predictions, which are beyond the frame of present analyses.

### 4.6 Amino Acids Inserted at Stops

A further useful comment by a reviewer suggested to investigate which amino acids are detected inserted at stops. Table 5 shows the distribution of amino acids inserted at stops for the various types of investigated non-canonical peptides, those translated from delRNAs, swinger RNAs, and from the latter, translated according to tetra- and pentacodons. These distributions overall resemble each other, hence biases for insertion of specific amino acid species at stops are explored for the sum of amino acids across all types of non-canonical peptides.

This distribution is compared to the distribution of amino acids in the 13 canonical, mitogenome-encoded proteins. Chi-square tests detect statistically significant positive biases for five amino acids, in decreasing order of bias: K, Q, C, D and E. The two first amino acids are identical to regular amino acids found most frequently inserted at stops by Aerni et al. [153]. This is a further indication that results presented here are not due to random false detections. In addition, this suggests that the mitochondrial system for translating stops resembles that found in bacteria, at least that from *Escherichia coli*.

### 4.7 Associations Between Independent Transcriptomic and Proteomic Data

A further important point raised by an anonymous reviewer relates to the origins of transcriptomic data, which are from patients with myeloid leukemia, versus the origins of proteomic data, which are from healthy patients. I previously discussed this issue for analyses of these data [26] along the following lines.

It is clear that if RNA and peptide data were obtained from the same cells, associations between RNA and peptide data would be strongest. The strength of the association would decrease if RNA and peptide were from the same tissues of the same individual(s), but not the same cells. Along that rationale, they would further decrease if RNA and peptide data were obtained from different individuals with similar backgrounds (e.g. all healthy).

Current analyses were done on data that were available to this author, in formats readily analyzable by available software, and for adequate quantities of data. The RNA and peptide data differ in cells, tissues, individuals and backgrounds. This means that statistically significant associations were repeatedly detected between RNA and peptide data despite a number of confounding factors that could mask RNA-peptide associations. The fact that associations between non-canonical RNAs and peptides were nevertheless repeatedly detected implies that the actual phenomenon is much stronger than evaluated in these suboptimal conditions.

A noisy background is more likely to mask than create statistically significant signals. In addition, noise would only occasionally create spurious associations, but associations were repeatedly detected. In fact, discrepancies between RNA and peptide origins explain why relatively few detected peptides map on detected RNAs. Nevertheless, these discrepancies could not prevent detecting associations between non-canonical RNAs and corresponding peptides.

## 5 Conclusions

- Unbiased analyses assuming random cleavage for tryptic data yield results biased towards tryptic peptides for peptides translated from non-canonical RNAs and along non-canonical translations. Results confirm previous trypsin-biased analyses that detected non-canonical peptides.
- Detected non-canonical RNAs associate with tryptic and non-tryptic peptides.
- Detected non-canonical peptides are overwhelmingly incompatible with translation according to the nuclear genetic code, and specifically match the mitochondrial vertebrate genetic code.
- Overall, results confirm translation of non-canonical RNAs (del- and swinger RNAs), and along expanded codons, in addition to detections of other types of non-canonical peptides, such as peptides translated from contiguous regular and swinger-transformed RNA [36].
- Proteomic analyses assuming random cleavage detect non-canonical peptides digested by natural proteolysis, expand proteomic coverage.

## Figures and Tables

**Table 1:** Abundances of residues at carboxyl extremities of non-canonical peptides detected by unbiased analyses. Analyses assume random cleavage of tryptic human mitoproteome. Peptides are translated from the del-, swinger-transformed human mitogenome, for codons expanded by 0–2 silent nucleotides. Column 1 indicates the residue. Columns 2, 6, 10 and 14 are numbers of detected peptides with residue indicated in 1, for each analysis assuming different transcription/translation (del-, swinger-, tetra- and pentacodon); 3, 7, 11 and 15 indicate total number of that residue in corresponding translations of the mitogenome; 4, 9, 12 and 16 indicate the bias of detecting peptides with that residue in carboxyl terminus position considering the total frequency of the residue in the corresponding translation of the mitogenome; 5, 9, 13 and 17 indicate numbers of peptides mapping on corresponding detected non-canonical RNAs. The two last lines compare results when merging tryptic vs other peptides, numbers of non-canonical peptides mapping on non-canonical RNAs are followed by expected numbers assuming random mapping.

1	2	3	4	5	6	7	8	9	10	11	12	13	14	15	16	17
AA	Del				Swinger				Swinger				Swinger			
	Tri	Genome	Bias	RNA	Tri	Genome	Bias	RNA	Tetra	Genome	Bias	RNA	Penta	Genome	Bias	RNA
A	10	8328	1.49	0	2	46,838	0.30	0	6	47,048	0.75	2	9	46,178	1.33	0
C	4	5680	0.87	0	0	22,697	0.00		5	22,836	1.29	0	0	22,108	0.00	0
D	1	4662	0.27	0	3	21,545	0.97	0	0	21,752	0	1	3	20,795	0.98	0
E	6	6281	1.18	0	4	24,954	1.12	0	7	25,290	1.63	0	4	24,200	1.13	0
F	5	7868	0.79	0	4	30,878	0.91	0	4	30,982	0.76	0	4	29,626	0.92	1
G	12	12,857	1.16	0	9	57,120	1.10	1	12	57,648	1.22	0	9	55,452	1.11	0
H	0	6578	0.00		4	22,775	1.23	0	7	22,836	1.80	1	5	21,803	1.56	0
IL	26	27,890	1.15	3	11	97,382	0.79	0	15	97,914	0.90	1	6	93,464	0.44	0
K	16	8142	2.43	2	10	30,603	2.29	0	14	30,982	2.65	0	14	29,608	3.22	2
M	6	7581	0.98	0	2	22,553	0.62	0	4	22,836	1.03	0	4	21,660	1.26	0
N	5	7723	0.80	0	7	26,449	1.85	0	6	26,660	1.32	0	5	25,427	1.34	1
P	9	12,857	0.87	3	6	57,489	0.73	0	7	57,648	0.71	0	7	55,452	0.86	0
Q	7	5647	1.53	2	6	24,126	1.74	0	13	24,206	3.15	0	4	23,422	1.16	0
R	9	5876	1.90	0	11	46,786	1.64	0	18	47,048	2.25	0	10	45,626	1.49	0
S	4	16,856	0.29	1	5	68,457	0.51	0	2	68,800	0.17	3	5	65,983	0.52	0
T	3	11,954	0.31	0	9	46,822	1.34	0	1	47,047	0.13	0	4	44,813	0.61	0
V	11	11,954	1.14	2	10	46,591	1.50	1	4	47,047	0.50	4	7	44,813	1.06	1
W	9	6838	1.63	1	3	23,960	0.88	0	4	24,206	0.97	0	1	23,118	0.30	0
Y	5	7630	0.81	0	0	23,238	0.00	0	2	22,836	0.51	0	4	21,364	1.28	0
Tot	148	183,202		14/6.31	106	741,263		2/3.02	127	745,622		12/3.38	105	714,912		5/2.94
Tryps	25	14,018	2.21	2/1.07	21	77,389	1.90	0/0.68	32	78,030	2.41	0/0.19	24	75,234	2.24	2/0.70
Others	123	169,184	0.90	12/5.24	85	663,874	0.90	2/2.34	95	667,592	0.84	12/3.19	81	639,678	0.85	3/2.24

**Table 2:** Pearson correlation coefficient r between abundances of non-canonical peptides detected by unsupervised proteomic analyses of trypsin-digested human mitochondrial proteomic MS/MS data and abundances of corresponding, previously detected non-canonical RNAs [22], [23]. Correlations are calculated separately for tryptic peptides (carboxyl extremity K or R) and other peptides. Non-canonical transcripts are del-and swinger-transformations of the human mitogenome, the latter translated along codons expanded by 0, 1 and 2 silent nucleotides. P values are one tailed, expecting positive correlations. Fisher's method for combining P values sums the − 2 × log Pi, where i runs from 1 to k. This sum follows a chi-square statistic distribution with 2 × k degrees of freedoms, where k is the number of Ps combined (here k = 4). Bold indicates statistical significance at P < 0.05.

Pearson r	Unbias Tryps		Other		
Transformation	r	P	r	P	All
Del	0.358	0.172	0.270	0.241	0.401
Swinger	**0.446**	0.016	0.171	0.217	0.253
Swinger tetra	0.109	0.310	0.099	0.327	0.143
Swinger penta	0.192	0.190	0.306	0.078	0.186
Combined chi	**17.41**	0.026	13.24	0.104	

**Table 3:** Bias in amino acid identity at the N-terminal (column 1) of the peptide after detected peptides, for unbiased analyses assuming random cleavage. Analysis search for peptides matching translations of the del- (columns 2–4) and swinger-transformed human mitogenome (columns 5–7), and translations of the swinger mitogenomes according to tetra- (columns 8–10) and pentacodons (columns 11–13). Columns 2, 5 , 8 and 11 incicate numbers of detections. ‘Genome’ (columns 3, 6, 9, 12) indicates abundances of that residue in the corresponding hypothetical translations of the complete mitogenome after transformations and non-canonical translations. Biases (columns 4, 7, 10, 13) do not resemble those for carboxyl-extremities of detected peptides (Table 1) and are less extreme. Overall they match random distributions around ‘1’, indicating lack of bias. This suggests that there is no or very little natural proteolysis with cleavage specificity related to the N-terminal of peptides after detected peptides.

1	2	3	4	5	6	7	8	9	10	11	12	13
AA	Del			Swinger			Swinger			Swinger		
	Tri	Genome	Bias	Tri	Genome	Bias	Tetra	Genome	Bias	Penta	Genome	Bias
A	8	8328	1.19	6	46,838	0.79	10	47,048	1.09	3	46,178	0.42
C	4	5680	0.87	3	22,697	0.82	6	22,836	1.34	8	22,108	2.31
D	6	4662	1.59	3	21,545	0.86	7	21,752	1.64	5	20,795	1.54
E	6	6281	1.18	8	24,954	1.98	7	25,290	1.41	2	24,200	0.53
F	5	7868	0.79	2	30,878	0.40	5	30,982	0.82	2	29,626	0.43
G	13	12,857	1.25	8	57,120	0.87	8	57,648	0.71	4	55,452	0.46
H	6	6578	1.13	4	22,775	1.09	4	22,836	0.90	2	21,803	0.59
IL	20	27,890	0.89	20	97,382	1.27	25	97,914	1.30	18	93,464	1.23
K	5	8142	0.76	4	30,603	0.81	5	30,982	0.82	4	29,608	0.86
M	5	7581	0.81	6	22,553	1.64	5	22,836	1.12	4	21,660	1.18
N	9	7723	1.44	2	26,449	0.47	6	26,660	1.15	3	25,427	0.75
P	11	12,857	1.06	5	57,489	0.54	12	57,648	1.06	11	55,452	1.27
Q	2	5647	0.44	4	24,126	1.24	4	24,206	0.84	4	23,422	1.09
R	3	5876	0.61	8	46,786	1.06	3	47,048	0.33	13	45,626	1.82
S	12	16,856	0.88	8	68,457	0.72	19	68,800	1.41	11	65,983	1.06
T	11	11,954	1.14	12	46,822	1.58	9	47,047	0.98	8	44,813	1.14
V	9	11,954	0.93	13	46,591	1.72	5	47,047	0.54	6	44,813	0.96
W	7	6838	1.27	2	23,960	0.52	2	24,206	0.42	2	23,118	0.55
Y	6	7630	0.97	2	23,238	0.53	4	22,836	0.90	2	21,364	0.60
Tot	148	183,202		120	741,263		146	745,622		112	714,912	

**Table 4:** Observed (column 4) and expected (column 5) numbers of detected non-canonical peptides compatible with translations according to each nuclear and mitochondrial vertebrate genetic codes. Predictions account for peptide length (mean length and standard deviation in columns 2 and 3), considering that translation of 60/64 (0.9375) codons is identical between these genetic codes. Results indicate strong biases against detection of peptides compatible with both genetic codes, showing that detected populations of peptides are specifically translated according to the mitochondrial vertebrate genetic code. This systematic bias excludes that detected non-canonical peptides have cytosolic origins.

1	2	3	4	5
Transformation	AAs	Sd	Obs	Exp
Del	18.28	5.96	23	48.75
Swinger tri	21.23	9.75	27	36.26
Swinger tetra	17.37	7.38	19	52.45
Swinger penta	17.37	7.79	23	40.40

**Table 5:** Distributions of amino acids inserted at stops in detected non-canonical peptides (columns Del, Swinger tri, Swinger tetra and Swinger penta), compared to the distribution of amino acids in canonical proteins encoded by the human mitogenome (Mito). Bias is the ratio between the frequency of the amino acid across all non-canonical peptides (column All) and its frequency in canonical proteins. P values are calculated using a chi-square test. Statistically significant results at P < 0.05 are underlined, and in bold when these are positive biases indicating greater than expected insertions at stop codons.

AA	Mito	Del	Swinger tri	Swinger tetra	Swinger penta	All peptides	Bias	P
A	225	1	10	7	1	19	0.65	0.062
C	22	3	3	3	0	9	3.15	**0.002**
D	66	2	5	10	0	17	1.98	**0.010**
E	88	5	9	4	2	20	1.75	**0.020**
F	216	3	2	4	1	10	0.36	0.0006
G	212	16	9	12	1	38	1.38	0.058
H	97	0	4	4	0	8	0.64	0.208
I,L	963	19	6	19	46	90	0.72	0.0006
K	95	18	22	22	11	73	5.92	**4 × 10**^**− 40**^
M	208	8	9	6	5	28	1.04	0.853
N	164	8	7	5	3	23	1.08	0.723
P	219	5	3	9	5	22	0.77	0.237
Q	90	18	7	8	10	43	3.68	**2 × 10**^**− 14**^
R	63	4	2	2	3	11	1.35	0.359
S	274	9	5	10	10	34	0.96	0.796
T	351	10	8	6	5	29	0.64	0.013
V	167	5	5	5	2	17	0.78	0.328
W	104	2	2	3	3	10	0.74	0.356
Y	135	2	1	7	4	14	0.80	0.414

## Appendix A Supplementary data

Supplementary material 1 Image 2

Supplementary material 2 Image 3
